# Novel *Gluconobacter oxydans* strains selected from Kombucha with potential postbiotic activity

**DOI:** 10.1007/s00253-023-12915-4

**Published:** 2023-12-29

**Authors:** Katarzyna Neffe-Skocińska, Ewa Długosz, Lidia Szulc-Dąbrowska, Dorota Zielińska

**Affiliations:** 1 Institute of Human Nutrition Sciences, Warsaw University of Life Sciences (WULS), Nowoursynowska St. 159, 02-776 Warsaw, Poland; 2 Institute of Veterinary Medicine, Warsaw University of Life Sciences (WULS), Nowoursynowska St. 159, 02-776 Warsaw, Poland

**Keywords:** Kombucha, *Gluconobacter oxydans*, Antimicrobial activity, Anticancer activity, Postbiotic

## Abstract

**Abstract:**

Gastric and colorectal cancer are among the most frequently diagnosed malignancies of the gastrointestinal tract. Searching for methods of therapy that complements treatment or has a preventive effect is desirable. Bacterial metabolites safe for human health, which have postbiotic effect, are of interest recently. The study aimed to preliminary assessment of the safety, antimicrobial, and anti-cancer activity of cell-free metabolites of *Gluconobacter oxydans* strains isolated from Kombucha beverages as an example of the potential postbiotic activity of acetic acid bacteria (AAB). The study material consisted of five AAB strains of Kombucha origin and three human cell lines (gastric adenoma—AGS, colorectal adenoma—HT-29, and healthy cells derived from the endothelium of the human umbilical vein—HUVEC). Results of the study confirms the health safety and functional properties of selected AAB strains, including their potential postbiotic properties. The best potential anticancer activity of the AAB cell-free supernatants was demonstrated against AGS gastric adenoma cells. The conducted research proves the postbiotic potential of selected acetic acid bacteria, especially the KNS30 strain.

**Key points:**

•*The beneficial and application properties of acetic acid bacteria are poorly studied.*

•*Gluconobacter oxydans from Kombucha show a postbiotic activity.*

•*The best anticancer activity of the G. oxydans showed against gastric adenoma.*

## Introduction

Beverage Kombucha is a combination of tea and sugar with microorganisms that are responsible for its characteristic health properties. The oxidative fermentation process is carried out by symbiotic cultures of microorganisms (Symbiotic Cultures of Bacteria and Yeast - SCOBY) forming the so-called “tea fungus.” The consortium consists of yeasts and acetic acid bacteria (AAB) and also few lactic acid bacteria (LAB). Studies have shown high content of compounds produced during oxidative fermentation in the Kombucha beverage, inter alia organic acids, polyphenols, and other bacterial and fungus metabolites. In addition, Kombucha contains water-soluble vitamins, primarily B vitamins and minerals. There are also reports where it is indicated that the Kombucha has an anticancer, anti-inflammatory, and antioxidant properties (Martínez Leal et al. 2018; Villarreal-Soto et al. 2019; Morales 2020).

*Gluconobacter oxydans* is often included acetic acid bacteria (AAB) in the Kombucha SCOBY consortium. *G. oxydans* is a gram-negative AAB belonging to the family *Acetobacteraceae*. The microorganism is an obligate aerobe, having a respiratory type of metabolism using oxygen as the terminal electron acceptor and brings about the incomplete oxidation of sugars, alcohols and acids. These strains are non-pathogenic towards man and animals (Gupta et al. 2001; Lynch et al. 2019).

Efficient oxidative metabolism of AAB concerns the production of compounds that can be widely used not only in the food industry. The variety of use of these bacteria is particularly illustrated by *G. oxydans*. AAB, particularly strains of the genus *Gluconobacter*, which have an enormous oxidative capacity, can be used for the oxidative conversion of d-sorbitol to l-sorbose, an important intermediate in the industrial production of l-ascorbic acid (vitamin C). AAB have the ability to produce polysaccharides, of which cellulose is the most common (La China et al. 2018). In addition to it, acetic acid bacteria also produce levan. It is obtained extracellularly from sugar substrates by bacteria such as *Gluconobacter*, *Komagataeibacter*, *Kozakia*, and *Neoasaia*. In industry, levan is used as an emulsifier, coloring and flavoring carrier, as well as a fat substitute. In addition, it has antioxidant and anti-inflammatory properties (Gomes et al. 2018). AAB may produce bacterial cellulose, which is characterized by its purity compared to plant cellulose (non-elimination of industrial costs associated with purification). Many other bacterial species also have the capacity to be cellulose-producing, but AAB has a high process efficiency, especially in the Kombucha beverage. Additionally, it has many desirable physicochemical characteristics such as high crystallinity, water absorption capacity, tensile strength, and biodegradability. Bacterial cellulose can be used as a gelling agent, food stabilizer and thickener, and wound healing agent (placed in dressings) and can also protect cells from UV radiation (Gullo et al. 2018). An exopolysaccharide produced alongside cellulose is levan, which is equally biodegradable and forms biofilms; it has a beneficial effect on the animal microbiome (Ua-Arak et al. 2017). Additionally, AABs are capable of synthesizing acetate, dextran, and mannan (Xu et al. 2019). In a study by Wichienchot (2005), glucooligosaccharides produced by *G. oxydans* NCIMB 4943 strain were shown to have prebiotic properties for human intestinal microbiota.

Despite numerous beneficial characteristics of ABB, these bacteria remain poorly studied. Identification remains another problem, but through genetic engineering methods, an increasing number of strains are being discovered. Therefore, it appears crucial to understand the mechanisms and beneficial properties of AAB. Conscious use of AAB during food production processes could contribute to the creation of products with favored characteristics. Such products would be the natural source of bacteria showing desired properties and could be applied for preventive purposes or as supplements during targeted pharmacological therapies.

Gastric and colorectal cancer are among the most frequently diagnosed malignancies of the gastrointestinal tract, especially among older males (Bray et al. 2018; Rawla and Barsouk 2018; Arafa et al. 2021; Abbasi et al. 2023). Over one million cases of gastric cancer are diagnosed each year around the world (Shayan et al. 2020; Bray et al. 2018). The main cause of gastric cancer is history of *Helicobacter pylori* infection, also an inappropriate lifestyle, smoking, and an incorrect diet, i.e., high consumption of table salt, smoked and pickled food, nitrosamines, and aflatoxins. In the case of colorectal cancer, epidemiological data are also not optimistic. The World Health Organization predicts that by 2030, this disease will cause approximately 17 million deaths (Rad et al. 2021). As a consequence, the burdened organism fighting the developing cancer is additionally weakened by the applied clinical treatment. The high frequency of gastric and colon cancers have incited researchers to look for novel and natural components as adjuncts to prevent its expansion (Abbasi et al. 2023). Therefore, any alternative related to the search for supportive treatment based on natural active ingredients, especially food and beverages with antioxidants properties, with probiotic or postbiotic components is worth for attention and further research The solution may be an optimized diet, including high consumption fresh fruit and vegetables, wholegrain bread, and vitamins C, A, and E, and especially fermented by beneficial starter culture microorganisms food product. Hence, the aim of the research described below became to preliminary assessment of the safety, antimicrobial, and anti-cancer activity of cell-free metabolites of *Gluconobacter oxydans* strains isolated from Kombucha beverages as an example of the potential postbiotic activity of acetic acid bacteria.

## Material and methods

### *Gluconobacter oxydans* strains

Five *Gluconobacter oxydans* strains (KNS30, KNS31, KNS32, K1, and K2) were isolated from a local Kombucha beverages (Mazovia, Poland). Strains KNS30-32 were from different batches of beverage than strains K1 and K2. All study microorganisms are part of the Warsaw University of Life Science Culture Collection and have been subjected to phenotypic and genotypic identification according to Neffe-Skocińska et al. (2019). DNA sequences are available in National Center for Biotechnology Information GenBank^®^ genetic sequence database (GenBank accession no: KNS30 - OQ594752; KNS31 - OQ594751; KNS32 - OQ597203). Strains KNS30-32 were accepted as a public deposit in the Collection of Industrial Microbial Cultures-Center for Microbiological Resources, Institute of Agricultural and Food Biotechnology-State Research Institute (https://www.ibprs.pl/en/) (IBPRS-PIB collection accession no: KNS30 - KKP 3997, KNS31 - KKP 3999, KNS32 - KKP – 3998).

### Cell-free supernatant (CFS) and cell-free neutralized supernatant (CFNS) preparation

The study AAB strains are stored in cryopreserved vial at − 80 °C. The process of reviving of bacterial strains was carried out in accordance to Neffe-Skocińska et al. (2019). Solid and liquid GC medium (glucose calcium carbonate) contained components required for adequate growth of acetic bacteria (0.3% peptone, 0.3% yeast extract, and 2% glucose). The ethanol (2%), which is the basic substrate for the formation of organic acids, was added to the media. In order to obtain and multiply the bacteria, 0.1 mL of material was taken and added to 5 mL of GC liquid medium. Cells of *G. oxydans* were cultivated in GC liquid medium intended for this group of microorganisms at 25 °C for 72 h. After this time, passage was carried out until the culture was reached at a density of approx. 10^7^ log/mL. Final step was to produce the pure post-culture supernatants. For this, cultures have been centrifuged (10,000 rpm per 5 min), and the supernatant has been separated from cell biomass. After this, the bacterial suspension was filtered through sterile syringe filters 0.22 µM (Qpore-Bionovo, Poland). That prepared CFS were used to obtain an antimicrobial activity properties of study strains. The pH value of CFS was KNS30 = 3.47; KNS31 = 3.72; KNS32 = 4.31; K1 = 3.99; K1 = 4.21; and K2 = 4.20. In order to determine the cytotoxic properties and the effect on the apoptosis process, cell-free neutralized supernatant (CFNS) was further investigated. In order to prepare the CFNS, the CFS were adjusted to pH 7.4 by addition of 2 M NaOH.

### Antibiotic susceptibility assay

Antibiotic susceptibility assays for study AAB strains to selected antibiotics were performed using MIC Strips (Liofilchem Diagnostici, Italy). The method consists in determining the Minimal Inhibitory Concentration (MIC) of an antibiotic against microorganisms on an impregnated paper strip with a defined gradient of concentration of a given bactericidal substance (μg/mL). Antibiotic susceptibility zones were measured in accordance with the guidelines provided by the manufacturer. Three independent experiments were performed.

### Antimicrobial activity assay

Standard ATCC (American Type Culture Collection) pathogenic microorganisms were used: *Listeria monocytogenes* ATCC 19111; *Listeria monocytogenes* ATCC 15313; *Salmonella* ATCC 13076; *Staphylococcus aureus* ATCC 25923. Each time, 50 µL of the pathogen suspension was pipetted into a tube containing 5 mL of M. Hinton liquid culture medium (BioMaxima, Poland). The operation was repeated for all ATCC strains. After this process was completed, tubes were incubated for 48 h at 37 °C.

The antimicrobial properties of AAB CFS were determined using the well diffusion method based on Ołdak et al. (2017), with own modification. Standard pathogenic microorganisms were surface plated onto M. Hinton poured agar plates by transferring 100 µL of liquid culture suspension. The next step was to cut the wells on each of the plates with the surface inoculation of the reference ATCC strains. The last step was to collect 200 µL of CFS and transfer it to a well in a plates. A total of 200 µL of each CFS from each AAB strain was transferred to a separate well, respectively. The plates were incubated for 48 h at 37 °C. The test was performed in three repetitions. The antimicrobial activity was calculated as follows (Ołdak et al. 2017):

$$\mathrm x\;=\;\mathrm D\;-\;\mathrm d,\;\mathrm{where}\;\mathrm D\;\mathrm{is}\;\mathrm{the}\;\mathrm{diameter}\;\mathrm{of}\;\mathrm{growth}\;\mathrm{inhibition}\;\mathrm{zone}\;\mathrm{and}\;\mathrm d\;\mathrm{is}\;\mathrm{the}\;\mathrm{well}\;\mathrm{diameter}\;(5.5\;\mathrm{mm}).$$

All results were classified by zone diameters and described in the following order: very low ( *x* < 4 mm), medium (*x* = 4–8 mm), high (*x* = 8–12 mm), and very high (*x* > 12 mm).

### Determination content of the organic acids in cell-free supernatant (CFS)

HPLC-UV analysis were conducted to determine organic acids, following the method by Švecová et al. (2015) with slight modifications. The analysis was performed using a Prominence HPLC system comprising an SIL-20A autosampler, a quaternary pump, degasser, column oven, and a PDA detector (SPD-M20A). Chromatographic separation utilized a Luna Omega Polar C18 column (3.0 µm; 4.6 × 250 mm) from Phenomenex with 100 mM potassium phosphate solution as the isocratic mobile phase at a flow rate of 1 mL/min for 26 min at 30 °C. The PDA detector simultaneously recorded data in the 200 to 600 nm range at a sample rate of 40 Hz, focusing on a wavelength of 210 nm with a 9-nm filter bandwidth to capture the elution of organic acids. Each extract was analyzed in triplicate, and the peaks were identified by comparison of the retention time and UV-vis spectroscopic data with authentic standards.

### Cell cultures

Human gastric adenocarcinoma (AGS) and colorectal (HT-29) cancer cell lines used in this study were obtained from ECACC (England). HT-29 cells were grown in McCoy’s 5A Medium (Sigma-Aldrich) and AGS in Ham’s F-12K (Kaighn’s) Medium (ThermoFisher), both supplemented with 10% fetal bovine serum, penicillin/streptomycin, and 2 mM L-glutamine. The umbilical vein endothelial cells (HUVEC) were obtained from Cell Applications, Inc. (USA) and used as a control. The culture was kept in the Endothelial Cell Growth Medium (Cell Applications, Inc. (USA), all-in-one ready-to-use medium). All cells were grown in T-75 flasks containing 20 mL of the appropriate medium in a humidified atmosphere with 5% CO_2_ at 37 °C. The cells were subcultured when the culture reaches 80% confluency.

### Cell viability assay

AGS, HT-29, and HUVEC cells were seeded in 96-well plates with 10^4^ cells per well in 80 μL of appropriate medium. Each time 24 h after seeding 20 µL of CFNS, fresh GC medium and taxol (7.5 nM) were added to wells, and the cultures were incubated for another 24 and 48 h. Additionally, corresponding controls contain medium and CFS, but no cells were prepared.

The CyQUANT^TM^ XTT Cell Viability Assay (Invitrogen by Thermo Fisher Scientific) was used according to the manufacturer’s protocol. Seventy microliters of XTT labeling mixture was added to wells and incubated for 4 h in 37 °C, 5% CO_2_. The absorbance was measured at 450 and 650 nm in BioTek Synergy H1 plate reader. OD values of each culture were divided by OD values of cell-free controls, and the results were calculated as signal/background ratio.

Three control variants were planned for this experiment. The control samples involved taxol (a chemotherapeutic compound against cancer cells), pure GC acetic acid bacteria culture medium (GC), and cell lines media. The cytotoxic effect of AAB metabolites was assayed after 24 and 48 h of incubation with selected cell lines. Three independent experiments were performed.

### Annexin V and propidium iodide (PI) staining and cytometric measurements

AGS, HT-29, and HUVEC cells were seeded in 24-well plates with 10^5^ cells per well in 800 μL of appropriate medium. Twenty-four hours after seeding 200 µL of CFNS, fresh GC medium and taxol (7.5 nM) were added to wells, and the cultures were incubated for another 48 h. Then, cells were rinsed with 1 mL of PBS and detached with trypsin-EDTA solution and centrifuged (200 × *G*, 5 min). Cells were prepared according to FITC Annexin V Apoptosis Detection Kit I (BD Pharmingen) protocol. Cell pellets were washed twice with cold PBS and suspended in 1X Binding Buffer and stained with FITC Annexin V and propidium iodide (PI) for 15 min at room temperature in the dark. Cells were then analyzed using LSR Fortessa flow cytometer (BD Bioscience).

An analogous arrangement of research variants was used as section 2.4. Three independent experiments were performed.

### Statistical analysis

The programs Microsoft Excel and GraphPad Prism 9 were used for the statistical analysis of the data. The mean were analyzed by the one-way ANOVA and Student *t*-methods. The results of studies on cell lines were compared to the control (GC—microbiology growth medium for AAB). All data are expressed as the mean ± standard deviation (SD) of triplicate measurements.

## Results

### Antibiotic susceptibility assay

Table 1 presents the results of the determination of the minimum inhibitory concentrations (MIC) of selected antibiotics against study *G. oxydans* strains. The obtained results were divided into the values inhibiting the growth of the study AAB strains and the values of total resistance (*R*) for tested antibiotic. The study showed that all *G. oxydans* strains showed very similar susceptibility to most of the 12 antibiotics used, including vancomycin. Antibiotic that did not inhibit the growth of all the AAB was penicillin (except KNS31). Complete resistance was also found for strain KNS30 in the case of sulfamethoxazole. The strains K1 and K2 showed complete resistance to three antibiotics (clindamycin, streptomycin, and penicillin), and for this reason, they cannot be considered completely safe for human health.

**Table 1:** Minimum concentration values inhibiting the growth of AAB strains on selected antibiotics

Antibiotic	AMP	C	CD	CN	E	K	P	S	TE	VA	SMX	CIP
MIC [µg/mL]	0–256										0–1024	0–32
AAB strain												
KNS30	16	1.5	12	12	96	1	*R*	1.5	0.75	96	*R*	0.75
KNS31	6	6	12	12	96	1	8	1.5	2	96	96	0.12
KNS32	2	3	48	32	128	1	*R*	1.5	0.75	96	96	0.75
K1	48	3	*R*	24	32	1	*R*	*R*	1.5	64	24	0.12
K2	48	3	*R*	24	48	1	*R*	*R*	1.5	64	96	0.12

Explanatory notes: *AMP*, ampicillin; *C*, chloramphenicol; *CD*, clindamycin; *CN*, gentamicin; *E*, erythromycin; *K*, kanamycin; *P*, penicillin; *S*, streptomycin; *SMX*, sulfamethoxazole; *TE*, tetracycline; *VA*, vancomycin; *CIP*, ciprofloxacin; *MIC*, minimum inhibitory concentration; *R*, total resistance of AAB strain to given antibiotic

### Antimicrobial activity assay

Table 2 presents the results of antimicrobial properties of metabolites of the *G. oxydans* strains. The pathogen most sensitive to AAB CFS was *Salmonella* (ATCC13076), where a very high antimicrobial effect was observed for all tested *G. oxydans* strains (except KNS32), whereas the most resistant pathogen bacteria to AAB CFS was *Listeria monocytogenes* ATCC15313 (except KNS30). The cell-free supernatant derived from the KNS30 strain showed the best growth-inhibiting properties of the all pathogens. Medium inhibitory effect of *G. oxydans* KNS30 metabolites were shown against *L. monocytogenes* ATCC19111; a very high effect was observed against *L. monocytogenes* ATCC15313, *Salmonella* (ATCC13076), and the strongest effect against *St. aureus* (ATCC25923). CFS showing the weakest antimicrobial properties are metabolites derived from strain *G. oxydans* KNS32. The zone of inhibition was shown only for *Salmonella* sp., but at the lowest inhibitory level.

**Table 2:** The inhibitory effect of *Gluconobacter oxydans* strains against pathogenic microorganisms

Pathogen	*Listeria monocytogenes* 111	*Listeria monocytogenes* 112	*Salmonella* ATCC13076	*Staphylococcus aureus* ATCC25923
AAB strain	Antimicrobial activity *x* [mm]			
KNS30	9.5 ± 0.02	23.5 ± 0.05	32.5 ± 0.02	16 ± 0.01
KNS31	8.5 ± 0.02	5.5 ± 0.02	18.5 ± 0.01	4.5 ± 0.02
KNS32	0	0	5.5 ± 0.05	0
K1	14.5 ± 0.12	3.5 ± 0.14	29.5 ± 0.12	5.5 ± 0.10
K2	14.5 ± 0.05	8.5 ± 0.08	21 ± 0.02	19.5 ± 0.06

Explanatory notes: low (*x* < 4 mm diameter), medium (*x* = 4–8 mm), high (*x* = 8–12 mm), and very high (*x* > 12 mm)

### Profile of organic acids in AAB CFS with potential postbiotic effects

Table 3 presents the results of chromatographic analysis of the organic acids composition of the study cell-free AAB supernatants. Metabolites from the group of organic acids of the study *G. oxydans*, that were identified, include gluconic acid, glucuronic acid, acetic acid, pyruvic acid, fumaric acid, and lactic acid. The study CFS of AAB contained three key organic acids for this species: gluconic, acetic, and glucuronic acids, respectively. The highest concentrations of gluconic acid were found for strains KNS30, KNS32, and KNS31, respectively. The concentration of acetic acid in tested CFS was in the range of 1.47–1.92 µg/g and was highest in case of KNS30, KNS32, and KNS31 strains. The presence of glucuronic acid in CFS of AAB was also observed, but the values were significantly lower. Other organic acids (pyruvic, fumaric, and lactic) were at very low level (0.08–0.01 µg/g) in the tested supernatants.

**Table 3:** The content of organic acids in AAB cell-free supernatant

AAB CFS	Kind of organic acid					
	Gluconic acid	Glucuronic acid	Acetic acid	Pyruvic acid	Fumaric acid	Lactic acid
	µg/g	µg/g	µg/g	µg/g	µg/g	µg/g
KNS30	2.00^aA^ ± 0.01	0.87^aB^ ± 0.02	1.92^aC^ ± 0.01	0.06^aD^ ± 0.00	0.02^aD^ ± 0.00	0.05^aD^ ± 0.00
KNS31	1.87^bA^ ± 0.00	0.87^aB^ ± 0.01	1.78^bC^ ± 0.01	0.09^aD^ ± 0.00	0.02^aD^ ± 0.00	0.08^aD^ ± 0.00
KNS32	1.91^aA^ ± 0.00	0.77^bB^ ± 0.00	1.62^cC^ ± 0.01	0.06^aD^ ± 0.00	0.01^aD^ ± 0.00	0.06^aD^ ± 0.00
K1	1.50^cA^ ± 0.01	0.05^cB^ ± 0.00	1.49^dA^ ± 0.01	0.05^aB^ ± 0.00	0.01^aB^ ± 0.00	0.04^aB^ ± 0.00
K2	1.49^dA^ ± 0.01	0.46^dB^ ± 0.00	1.47^dA^ ± 0.01	0.05^aC^ ± 0.00	0.01^aC^ ± 0.00	0.05^aC^ ± 0.00

Explanatory notes: in the same column, numbers followed by different lowercase letters and by different capital letters in the same row represent significant differences between the samples in the Student* t*-test (*P* < 0.05); *n* = 3

### Cytotoxicity assay

**Fig. 1:**
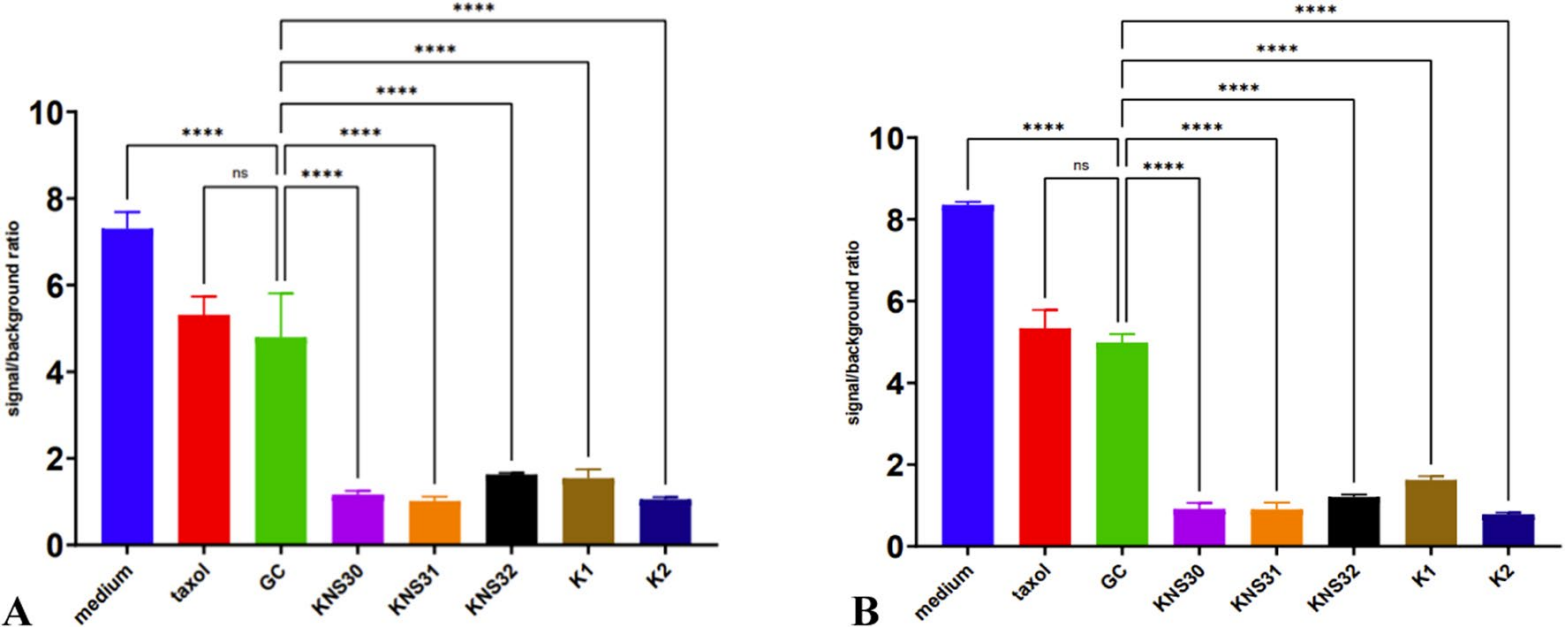
XTT assay of different cell-free supernatants of *G. oxydans* strains on AGS cancer cell line after **A** 24-h and **B** 48-h treatment. ****P < 0.0001; ns, no statistical differences

**Fig. 2:**
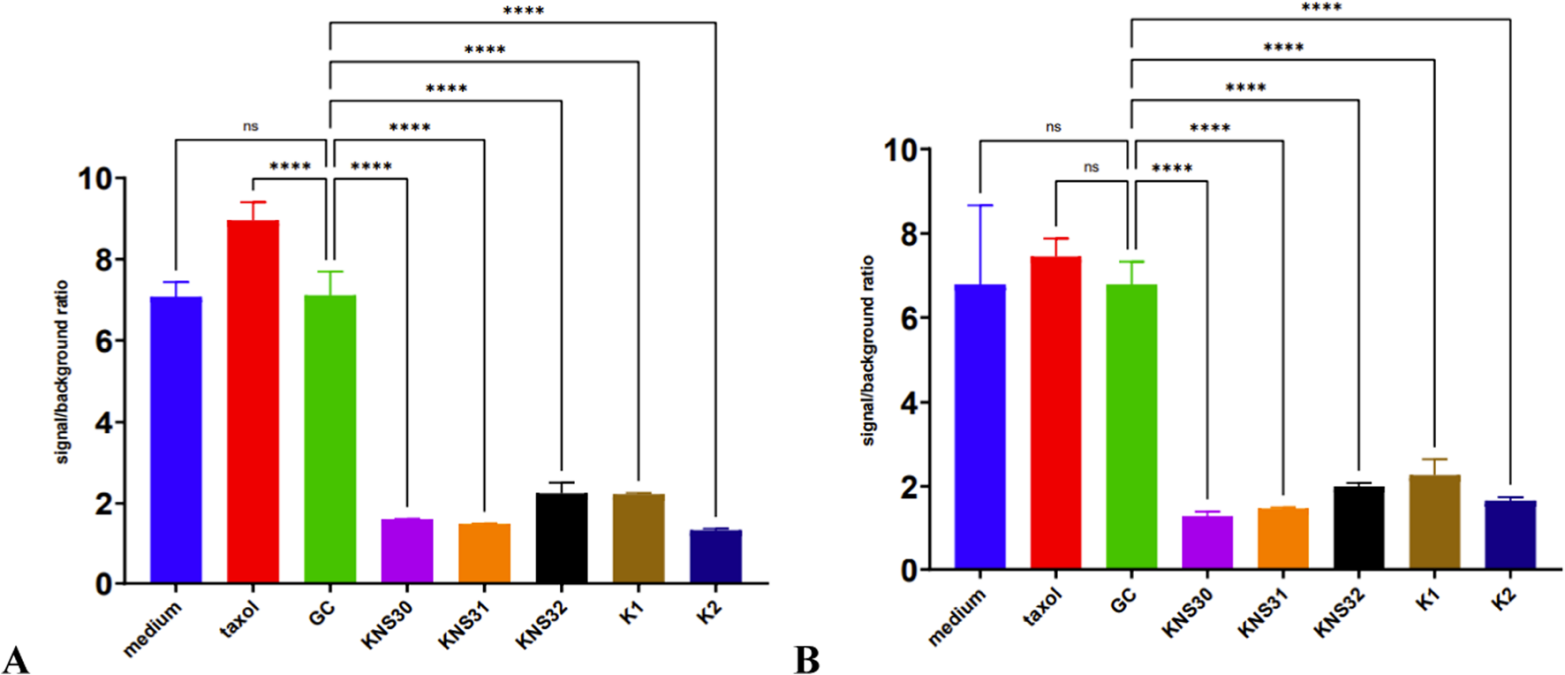
XTT assay of different cell-free supernatants of *G. oxydans* strains on HT-29 cancer cell line after **A** 24-h and **B** 48-h treatment. ****P < 0.0001; ns, no statistical differences

**Fig. 3:**
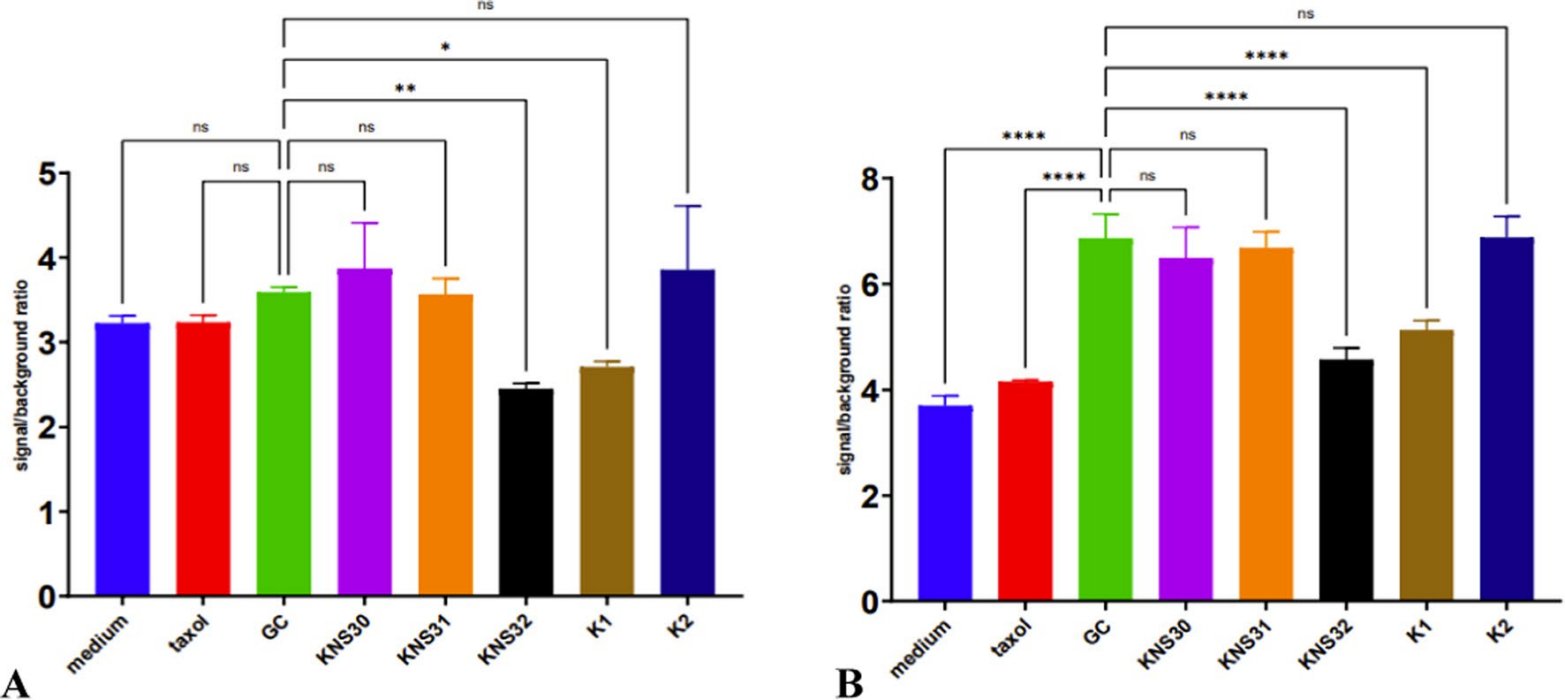
XTT assay of different cell-free supernatants of *G. oxydans* strains on HUVEC health cell line after **A** 24-h and **B** 48-h treatment. *P < 0.05, **P < 0.01, and ****P < 0.0001; ns, no statistical differences

Figures 1 and 2 present the impact of various AAB CFNS on the viability of human gastric and colon cancer cells. Figure 3 shows the impact of CFNS on the viability of HUVEC lines, as a reference for the safety of study metabolites in relation to human healthy cells. At this stage of the in vitro experiment, neutralized supernatants were used to eliminate the effect of low pH on the study human cells from individual lines and provide them with optimal growth conditions.

The strongest cytotoxic effect was observed in the case of AGS cancer line in comparison to other tested cell lines (Fig. 1). The reference in the statistical analysis was the microbial culture medium for acetic acid bacteria (GC). The addition of culture medium for acetic acid bacteria (GC) significantly reduced the viability of cancer cells, which could be due to the presence of ethanol not yet metabolized by AAB in this medium. This result was comparable to the effect induced by the addition of taxol, which is a well-known chemotherapeutic agent. CFNS from all five AAB strains further decreased the viability of AGS cells (*P* < 0.0001). The cytotoxic effect of these supernatants increased slightly with the incubation time, and after 48 h, the survival rate of the tumor cells was lower.

Colorectal cancer cells of the HT-29 line were characterized by greater resistance to the cytotoxic effect of taxol. However, the cytotoxic effect of AAB culture supernatants was much stronger than that of the chemotherapeutic drug and similar to that observed in AGS cells. After incubation, the significant differences were noted between the GC control and all five AAB supernatants.

HUVEC line was used to check whether the analyzed AAB supernatants did not have a negative effect on normal human cells. After 24 hours of incubation, KNS32 and K1 CFS showed a statistically significant cytotoxic effect comparing to GC (Fig. 3A). An interesting observation was made 48 h of incubation, when the viability of HUVECs incubated with GC was significantly higher comparing to medium and taxol controls. Again, KNS32 and K1 supernatants decreased cell viability comparing to GC (Fig. 3B).

### Quantification of apoptosis of cancer cell lines

**Fig. 4:**
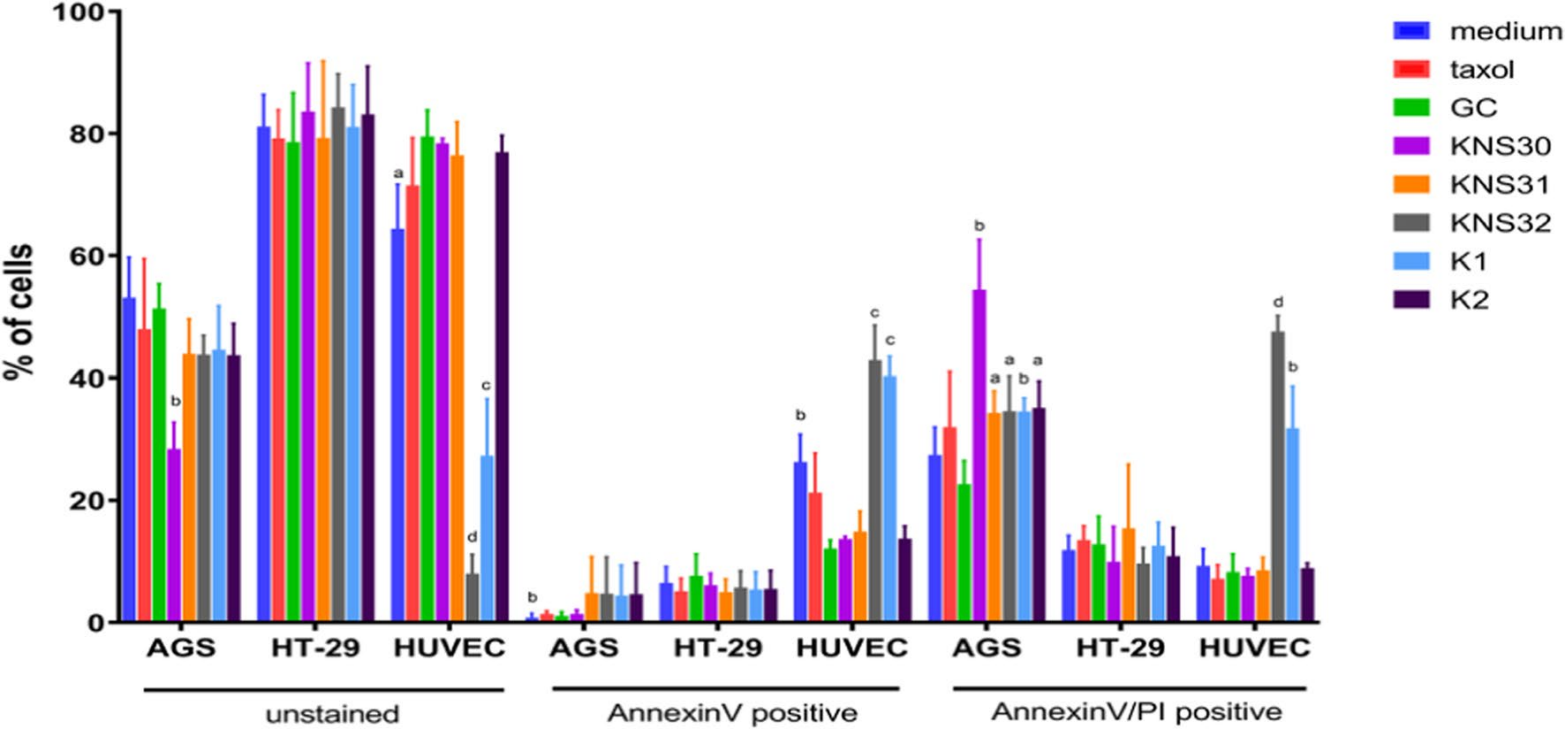
Apoptotic effects of cell-free neutralized supernatants of *G. oxydans* strains on AGS, HT-29, and HUVEC cell lines after 48 h of incubation process. The apoptosis rates with data as means mean value ± SD (*n* = 3). Unstained—live cells; annexin V positive—early apoptotic cells; annexin V/PI positive—late apoptotic and necrotic cells. *Statistically significant differences (a—*P* < 0.05; b—*P* < 0.01; c—*P* < 0.001; d—*P* < 0.0001 P) compared to GC medium

**Fig. 5:**
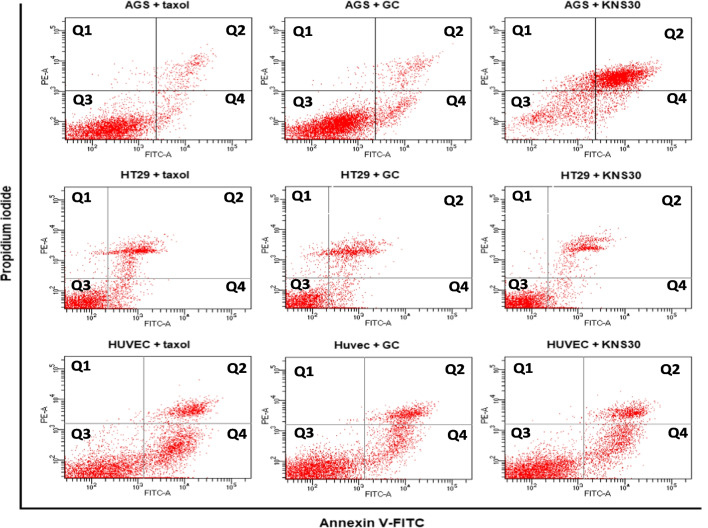
Representative examples of raw flow cytometry figures of apoptotic effects of cell-free neutralized supernatants of *Gluconobacter oxydans* KNS30 on AGS, HT-29, and HUVEC cell lines. Q2, late apoptotic/necrotic cells—annexin V and PI positive; Q3, early apoptotic cells—annexin V positive; Q4, live cells—unsatined

The flow cytometry method was used to quantitatively determine apoptosis in study cell lines treated with AAB CFNS for 48 h (Fig. 4). All cell-free supernatants of *G. oxydans* strains showed differential effects on AGS cancer cells. Only CFNS from KNS30 culture significantly decreased the number of live (unstained) AGS cells, whereas all five analyzed AAB supernatants increased the number of late apoptotic/necrotic (annexin V and PI positive) cells. On the contrary, KNS30 metabolites present in post-culture medium had no effect on normal endothelial cells, which, in turn, were significantly affected by KNS32 and K1 supernatants. In case of these two CFNS, the number of live HUVEC cells was significantly lower, and the number of early apoptotic (annexin V positive) and late apoptotic/necrotic cells (annexin V/PI positive) was higher. No effect was observed in case of HT-29 cells. Figure 5 shows the raw flow cytometry data representing the effect induced by KNS30 supernatant on all analyzed cell lines comparing to GC and taxol controls.

## Discussion

There are a number of aspects that newly isolated strains must meet. A newly notified taxonomic entity is initially evaluated considering aspects such as the associated body of knowledge, history of apparent safe use in food, scientific literature, and clinical aspects and industrial use. New strains as candidates for starter preparations for food fermentation and as potential probiotics or postbiotics, therefore, must obtain the status of safe for use, and also come from traditional food ingredients. They should also belong to a species defined as safe, e.g., *Gluconobacter* or *Komagataeibacter* (Lynch et al. 2019). The characterization of the new AAB strains began with determining the initial safety of the tested microorganisms by determining their source of origin, resistance to selected antibiotics and antimicrobial profile. The characterization of the new AAB strains began with determining the initial safety of the tested microorganisms by determining their source of origin, resistance to selected antibiotics and antimicrobial profile. The source of origin of the study AAB strains was the Kombucha beverage. Additionally, *G. oxydans* is included in the Qualified Presumption of Safety (QPS) list, the official document of European Food Safety Authority (EFSA), which is equivalent to the American Generally Recognized as Safe (GRAS) list (Lynch et al. 2019). Therefore, the first safety criterion was fulfilled. Another safety criteria for the study AAB strains presented below are antibiotic susceptibility assay. The limits of antibiotics susceptibility test included in particular for pathogenic microorganisms and are interpreted using appropriate breakpoints, according to recommendations of the American CLSI (Clinical Laboratory Standards Institute) or European EUCAST (European Committee on Antimicrobial Susceptibility Testing 2018) (Abbey and Deak 2019). There are no specific guidelines for non-pathogenic bacteria, including acetic acid bacteria. However, results of the obtained studies can be an important basis for diagnosing antibiotic resistance of new and safe functional strains of acetic acid bacteria. The last study criterion of new AAB strains was their antimicrobial activity. The obtained results indicated that all study AAB strains showed a good antimicrobial effect. However, the best growth-inhibiting properties against of the all study pathogens was demonstrated from *G. oxydans* KNS30 cell-free supernatant, also against *Listeria monocytogenes* ATCC15313. Correlations between the antimicrobial and antioxidant properties were related to the total phenolic contents and have been known for a long time.

The type of metabolites produced is a strain-dependent feature. AAB during their metabolic transformations can produce various organic compounds, which depends on the type of bacteria. These transformations occur as a result of cyclic series of biochemical reactions, including the citric acid cycle, tricarboxylic acids, and the Krebs cycle. The metabolism of most known *Acetobacter* sp. is characterized by the production of acetic acid from ethanol due to the presence of alcohol dehydrogenase and aldehyde dehydrogenase. In the Krebs cycle, bacteria of the *Acetobacter* sp. are also able to metabolize organic acids, including acetic, citric, fumaric, lactic, malic, pyruvic, and succinic acids. Different metabolic pathways occur in bacteria of the *Gluconobacter* sp. The metabolism of *G. oxydans* is based on the ability to incompletely oxidize a wide range of carbohydrates and alcohols (Lynch et al. 2019; Antolak et al. 2021). The result of *G. oxydans* metabolic activity are vitamin C, gluconic acid, glucuronic acid, and D-sacharic acid 1,4-lactone (DSL) synthesis and involved in the conversion of phenolic compounds. For example, glucuronic acid facilitates the bioavailability of polyphenols by conjugating them and facilitating their transport (Antolak et al. 2021). In conclusion, the metabolic activity of various AAB strains allows to obtain products characterized by various composition: the content of organic acids, vitamins, enzymes, and antioxidant activity, which among the health-promoting properties for humans has an antimicrobial effect. The conducted study showed that the tested bacteria produce significant amounts of gluconic acid, glucuronic acid, as well as acetic acid.

The study bacteria have high resistance to low pH and show sensitivity to antibiotics. In conclusion, bacterial species used in this study was chosen because source of their origin is the beverage kombucha; it has no pathogenicity to humans and animals; additionally, genus *Gluconobacter oxydans* is widely used in both food and biotechnology industries.

The next stage of the research was to draw attention to the beneficial and postbiotic properties of the study AAB strains, including their potential anticancer activity. Chemotherapy and radiotherapy are currently one of the basic tools to inhibit the development of cancer cells in the human body. Both therapies have a therapeutic effect, but they can also cause many adverse effects in human organs and tissues. Successful cancer treatment requires a constant search for innovative drugs with a low cytotoxic effect for all human body. The use of unpurified and unprocessed bacterial metabolites with health effects, including postbiotics with anti-carcinogenic activity, is described in many scientific papers (Bedada, et al. 2020; El-Deeb et al. 2018; Abbasi et al. 2023). Most of the research focuses on tumor cell lines like a HT-29 or Caco-2 and on the effect of probiotic lactic acid bacteria of the genus *Lactobacillus* and *Bifidobacterium* or fungi *Saccharomyces cerevisieae* var. *boulardii* on the prevention and treatment cancer (Lee et al. 2015; Wang et al. 2019; Abbasi et al. 2023). Interestingly, studies of dead cells of lactic acid bacteria or fungi with probiotic properties indicate that dead probiotic administration at high dose reduced a number of tumors considerably compared with pure live probiotic (Bedada et al. 2020; Lee et al. 2015). Only Haghshenas et al. (2015) and Aghazadeh et al. (2017) reported anticancer effect induced by potential probiotic acetic acid bacteria for *Acetobacter syzygii*. In the current study, AAB metabolites did not show significant cytotoxic effects on treated health human cells. On the other hand, taxol (a known chemotherapeutic drug) showed higher cytotoxic effects than the study supernatants of *G. oxydans* strains.

Determining the cytotoxicity of bacterial metabolites in relation to the study cell lines is not sufficient. An important aspect is the type of cell death that occurs under the influence of a factor that inhibits their growth and survival, as well as the effect of CFNS on the condition of health human cells. Apoptosis, called programmed cell death, is a physiological process that determines the proper functioning of the body, ensuring its homeostasis. This type of cell death is based on a controlled process of nuclear disintegration, chromatin condensation, and DNA fragmentation. The process is controlled by caspases (Chen et al. 2020). Thus, apoptosis is a desirable and beneficial process from the point of view of human health, in contrast to necrosis, which is responsible for the formation of inflammation in the body, through the random degradation of DNA and aggregation of chromatin (Chen et al. 2020; Abbasi et al. 2023). The study quantification of apoptosis of cell lines demonstrated that selected AAB strains post-culture mediums have potential anticancer properties, where the best effect was demonstrated on AGS in contrast to HT-29 cell lines. Research confirms the numerous metabolic capabilities of acetic acid bacteria, including their participation in the synthesis of organic acids and vitamin C (Shinjoh et al. 2016; Lynch et al. 2019). The most important products of *G. oxydans* metabolism are glucuronic acid (GlcUA) and his derivative D-saccharic acid 1.4-lactone (DSL). DSL inhibits enzyme glucuronidase, which is related with cancer genesis (Antolak et al. 2021). It is a component derived from D-glucaric acid (a product of metabolism) that in turn exhibits detoxification and antioxidant properties and demonstrates an ability to reduce oxidative damage (Saluk-Juszcak et al. 2008). DSL can also inhibit glucosidase activity and thus facilitate glucuronic acid removal of toxic substances, including carcinogens (PAHs, some nitrosamines, aromatic amines, and fungal toxins), some cancer promoters (steroid hormones), and hepatotoxins (acetaminophen), and inhibit the apoptotic death of pancreatic β-cell (Wang et al. 2019; Antolak et al. 2021). In addition, they may modulate estradiol and other steroid hormone levels and their excretion as glucuronides (Li et al. 2017; La China et al. 2018). It is also involved in the elimination of endobiotics and participates in the increased bioavailability of polyphenols. GlcUA is important in relation to the biotransformation and protection of fatty acids against lipid peroxidation, which is considered as a factor increasing the risk of developing certain pathologies, such as atherosclerosis, kidney damage, or Parkinson’s disease (Martinez Leal et al. 2018). Moreover, AAB strains have the ability to produce gluconic acid (GA), which improves the sensory properties of products, is a preservative, quality parameter of food products, and used as gluconate in the pharmaceutical industry for the production of supplements used in treatment of hypocalcemia and anemia (García-García et al. 2017). Thus, taking into account obtained results, these Kombucha origin acetic acid bacteria-secreted metabolites, especially of KNS30 and KNS31 strains, may be primarily proposed as effective for supporting the treatment of gastrointestinal cancers and, moreover, with potentially postbiotic effects.

According to the current definition of probiotics (Hill et al. 2014), acetic acid bacteria cannot be classified into this group, because the AAB strains are not a natural microbiota of human gastrointestinal truck and cannot adhere to the epithelial. However, the postbiotics of AAB may be beneficial for human health. According to the International Scientific Association for Probiotics and Prebiotics (ISAPP) consensus statement, postbiotics are a “preparation of inanimate microorganisms and/or their components that confers a health benefit on the host” (Salminen et al. 2021). According to the next statement of ISAPP, postbiotic products are derived from microorganisms, but do not have to be a probiotic derivative. Another ISAPP definition state that isolated and purified metabolites cannot be postbiotics. However, the separated and unprocessed supernatants from the bacterial biomass are postbiotics (Vinderola et al. 2022). These definitions fit into the study cell-free supernatants of acetic acid bacteria. Additionally, postbiotics or another term “paraprobiotics” have been used to describe non-viable microorganisms or bacterial-free extracts that may provide additional benefits to the host through their bioactive qualities (Karbowiak et al. 2022). These terms are currently assigned to lactic acid bacteria or selected fungi. On the basis of the obtained research results, it is worth paying attention to AAB, which qualify very well for the definition of postbiotics and a good alternative in this topic to lactic acid bacteria (Haghshenas et al. 2015; Lynch et al. 2019; Antolak et al. 2021). Fermented drink Kombucha is described by many authors as a health-promoting product with a wide spectrum of activity, including anti-cancer effect, among other, by the presence of DSL (Wang et al. 2019). An important aspect from the point of view of health-promoting properties of Kombucha tea is its potential probiotic or postbiotic nature, which results from the presence of living microorganisms and their metabolites Sengun and Kirmizigul (2020).

In conclusion, the conducted research confirms the health safety and functional properties of selected AAB strains, including their potential postbiotic properties. The study cell-free supernatants of *Gluconobacter oxydans* strains were showed a different ability to stimulate the death process of two human cell lines with neoplastic features and a non-cancerous cell line. The potential anticancer activity of the AAB cell-free supernatants, in particular the *G. oxydans* KNS30 strain, was demonstrated against AGS gastric adenoma cells. The conducted research proves the postbiotic potential of selected acetic acid bacteria, especially the KNS30 strain. The presented research will be continued due to identify the other specific metabolites which are health beneficial and may also be responsible for the observed effect.

## Data Availability

The data generated during the current study are available from the corresponding author on reasonable request.
